# Benzyl trichloroacetimidates as derivatizing agents for phosphonic acids related to nerve agents by EI-GC-MS during OPCW proficiency test scenarios

**DOI:** 10.1038/s41598-022-25710-4

**Published:** 2022-12-09

**Authors:** Alagu Subramanian, José A. Rosales, Roald N. Leif, Carlos A. Valdez

**Affiliations:** 1 Forensic Science Center, Lawrence Livermore National Laboratory, Livermore, CA USA; 2 Nuclear and Chemical Sciences Division, Lawrence Livermore National Laboratory, Livermore, CA USA; 3 Biosciences and Biotechnology Division, Lawrence Livermore National Laboratory, Livermore, CA USA; 4 NNSA-MSIIP Summer Fellow, University of Texas, El Paso, TX USA; 5 Global Security Directorate, Lawrence Livermore National Laboratory, Livermore, CA USA; 6 Lawrence Livermore National Laboratory, 7000 East Avenue, L-090, Livermore, CA 94550 USA

**Keywords:** Chemistry, Analytical chemistry, Mass spectrometry

## Abstract

The use of benzyl trichloroacetimidates for the benzylation of phosphonic acid nerve agent markers under neutral, basic, and slightly acidic conditions is presented. The benzyl-derived phosphonic acids were detected and analyzed by Electron Ionization Gas Chromatography–Mass Spectrometry (EI-GC–MS). The phosphonic acids used in this work included ethyl-, cyclohexyl- and pinacolyl methylphosphonic acid, first pass hydrolysis products from the nerve agents ethyl *N*-2-diisopropylaminoethyl methylphosphonothiolate (VX), cyclosarin (GF) and soman (GD) respectively. Optimization of reaction parameters for the benzylation included reaction time and solvent, temperature and the effect of the absence or presence of catalytic acid. The optimized conditions for the derivatization of the phosphonic acids specifically for their benzylation, included neutral as well as catalytic acid (< 5 mol%) and benzyl 2,2,2-trichloroacetimidate in excess coupled to heating the mixture to 60 °C in acetonitrile for 4 h. While the neutral conditions for the method proved to be efficient for the preparation of the *p*-methoxybenzyl esters of the phosphonic acids, the acid-catalyzed process appeared to provide much lower yields of the products relative to its benzyl counterpart. The method’s efficiency was tested in the successful derivatization and identification of pinacolyl methylphosphonic acid (PMPA) as its benzyl ester when present at a concentration of ~ 5 μg/g in a soil matrix featured in the Organisation for the Prohibition of Chemical Weapons (OPCW) 44th proficiency test (PT). Additionally, the protocol was used in the detection and identification of PMPA when spiked at ~ 10 μg/mL concentration in a fatty acid-rich liquid matrix featured during the 38^th^ OPCW-PT. The benzyl derivative of PMPA was partially corroborated with the instrument's internal NIST spectral library and the OPCW central analytical database (OCAD v.21_2019) but unambiguously identified through comparison with a synthesized authentic standard. The method’s MDL (LOD) values for the benzyl and the *p*-methoxybenzyl pinacolyl methylphosphonic acids were determined to be 35 and 63 ng/mL respectively, while the method’s Limit of Quantitation (LOQ) was determined to be 104 and 189 ng/mL respectively in the OPCW-PT soil matrix evaluated.

## Introduction

**Figure 1:**
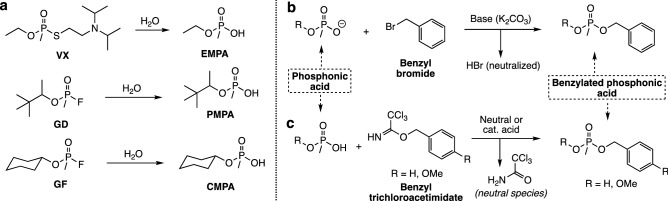
(**a**) Hydrolysis of the organophosphorus-based nerve agents VX, GD and GB to yield ethyl methylphosphonic acid (EMPA), pinacolyl methylphosphonic acid (PMPA) and cyclohexyl methylphosphonic acid (CMPA) respectively. Benzylation approaches for phosphonic acids using (**b**) benzyl bromide in the presence of a base and (**c**) using benzyl and *p*-methoxybenzyl trichloroacetimidates under neutral, basic or acidic conditions.

Recently, the deliberate use of organophosphorus-based nerve agents (OPNAs) in successful and failed assassination attempts in the world^1–3^, has resulted in a resurgence of research efforts towards the development of medical countermeasures against these lethal chemicals^4–6^, methods for their decontamination^7,8^ and more effective, alternative ways for their analysis and detection by various analytical methods^9,10^. Within the umbrella of OPNAs, the G- and V-series agents represent one of the most commonly employed historically within as well as outside military conflicts. Three important world events that illustrate their use against civilian targets are the use of sarin during the Tokyo subway system attack^11,12^, the Ghouta chemical attack in Syria^13,14^ and the use of VX in the assassination of Kim Jong Nam at the Kuala Lumpur airport in Malaysia^15^. Nerve agents may be detected by GC–MS means in their intact form, as they are liquids at ambient temperature and thus volatile when subjected to the high temperatures in the instrument. Now, even though some nerve agents enjoy more pronounced stability profiles than others, the mechanism by which all these degrade, under basic or oxidative conditions, is fairly predictable^16^. Consequently, all the OPNAs belonging to the G- and V-series undergo hydrolysis that is characterized by scission of the P-F (e.g., GD, GF) or P-CN (e.g., GA) bonds in G-based agents while most of the hydrolytic pathway for the V-series agents (e.g., VX, VR) will involve scission of the P–S bond^16–18^. The hydrolysis event leads to the formation of methylphosphonic acid half-esters that due to their immediate relationship to the nerve agent have become one of the most studied species in the chemical warfare analysis field by GC–MS^19,20^ (Fig. 1a) as well as in the chemical attribution signature analysis of OPNAs by various analytical methods^21–24^. However, direct detection of these hydrolysis products is troublesome due to their low volatility, reason why several methods for their derivatization have been developed that include silylation^25,26^ and alkylation in the form of methylation^27–30^ and benzylation^31,32^. These methods have proven to be essential chemical tools in the field of GC–MS and OPNA degradation products analysis and as such have enjoyed numerous research ventures into the development of efficient derivatization agents for their successful GC–MS analysis^16^. One method in particular, benzylation, has become a staple derivatization reaction for phosphonic acids providing adducts that bestow the phosphonic acid with a higher molecular weight and thus longer retention time in the column as well as enhanced volatility^33^. The established method for the introduction of the benzyl group involves the use of a base, usually sodium or potassium carbonate, to deprotonate the phosphonic acid followed by reaction with a benzyl halide (e.g*.*, benzyl bromide) with overnight heating at 70 °C^34^ (Fig. 1b). This reaction is efficient and works well under basic conditions which could create some problems when dealing with base-sensitive species present in a matrix that could be related to another chemical warfare agent of interest. To this end, a protocol for the benzylation of phosphonic acids involving neutral or slightly acidic conditions could provide analysts with an alternative way of introducing the benzyl moiety into phosphonic acids. The method herein introduced employs two reagents, benzyl- and *p*-methoxybenzyl-2,2,2-trichloroacetimidate, for the formation of benzyl esters of phosphonic acids related to OPNAs (Fig. 1c).

## Results and discussion

Derivatizations in the form of alkylations such as methylations^35–37^ and benzylations have become common practice in the analysis by GC–MS of species that in their native form are marginally detectable by the technique. As these two alkylation reactions have received the most attention, it is no surprise that most instruments libraries like NIST^38,39^ and the OPCW central analytical database (OCAD)^40^ have amassed a large collection of methylated and benzylated phosphonic acids that are crucial in the routine and real case scenario identification of these nerve agent degradation products. In general, benzylation of phosphonic acids for their subsequent analysis by GC–MS methods are carried out under basic conditions and using a benzyl halide (e.g., benzyl bromide or pentafluorobenzyl bromide)^33,41,42^. The base employed in these instances could be organic or inorganic in nature, both leading to the formation of the benzylated phosphonic acid species in good yields. The added benefit of using an inorganic base, such as potassium carbonate, is that it provides cleaner mass spectra in contrast to the organic ones, such as triethylamine, that could interfere with key signals since they are typically used in excess during the derivatization. As an alternative method for the installment of the benzyl group we turned to the benzyl trichloroacetimidates^43,44^ which are activated forms of benzyl alcohol that can react in similar fashion to benzyl bromides in the benzylation of substrates but in this case under neutral to acidic conditions. Another attractive feature of trichloroacetimidates is that they are in most cases stable liquids at ambient temperature making their use in derivatizations convenient as weighing of solids is eliminated. The three phosphonic acids chosen for the studies in this work were ethyl-, cyclohexyl- and pinacolyl methylphosphonic acids which are the hydrolysis products for the nerve agents ethyl *N*-2-diisopropylaminoethyl methylphosphonothiolate (VX), cyclosarin (GF) and soman (GD) respectively.

### Optimization for the benzylation

**Figure 2:**
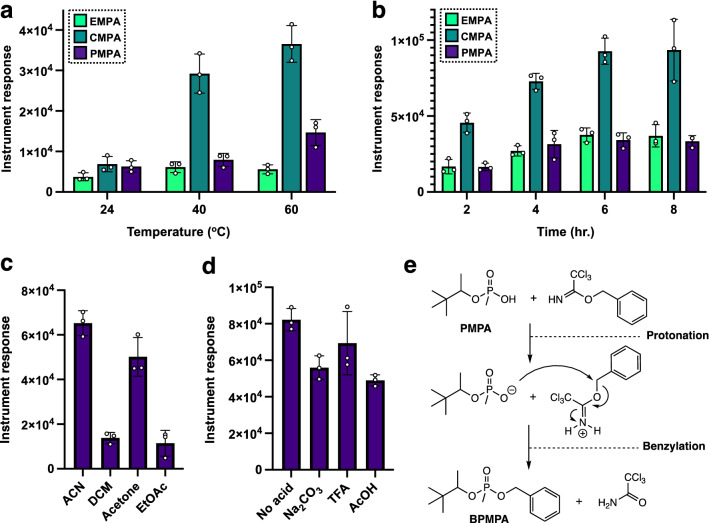
Optimization reactions, average (n = 3) peak areas (± the standard deviation) for the benzylated phosphonic acid after the acid (0.8 μmol, [100 ppm]) is treated with the trichloroacetimidate (200 μL, excess) in acetonitrile (ACN, 1 mL), that included (**a**) the effect of temperature on the derivatization yields similar results for mild heating (40 °C) and heating to 60 °C for 4 h; (**b**) time for the reaction where a maximum accumulation of the three benzylated products can be observed at ~ 4 h; (**c**) studies on just PMPA reveal that acetonitrile and acetone are optimal solvents for the derivatization over DCM and EtOAc; (**d**) effect of no additives (‘neutral’), base (20 mg, excess) and catalytic acid (TFA and AcOH, 0.08 μmol (10 mol% relative to phosphonic acid) on the reaction demonstrates that the reaction works well under all these conditions; (**e**) proposed mechanism for the reaction when no acid or base is added, with the phosphonic acid acting as the activating species for the trichloroacetimidate.

One of the first steps in demonstrating the feasibility of using benzyl trichloroacetimidates for the derivatization of phosphonic acids was to find the most optimal conditions for the reaction. After determining the feasibility of the derivatization using similar conditions (ACN/60 °C/2 h) to other derivatization protocols (e.g., BSTFA-mediated silylation), the temperature for the reaction became the first parameter to be evaluated. It was found that the reaction yielded the most product for each phosphonic acid evaluated when it was carried out at 60 °C, however, substantial benzyl ester products of each phosphonic acid were found to accumulate at 40 °C as well (Fig. 2a) at a set time of 2 h. After the temperature (60 °C) was determined to be optimal for the reaction when carried out for only 2 h, the reaction’s yield for each benzyl ester for each phosphonic acid with respect to time was determined. The times evaluated for optimization were 2, 4, 6 and 8 h. It was found that the reaction yielded significant benzyl ester product for each phosphonic acid after 4 h and that the accumulation of these products did not experience a larger increase afterwards upon extended reaction time (up to 8 h) (Fig. 2b). Therefore, this data shows that an initial GC analysis can be done as early as 4 h into the derivatization and depending on the analyte concentration, extended periods of time for the derivatization (up to 8 h) can be applied to increase the yield of the benzylated phosphonic acids. After these first two parameters were optimized (temperature and time), we turned our attention to evaluating the effect of the solvent used in the reaction as well as the impact of conducting the reaction under neutral, basic and added catalytic acid conditions. The last two parameters set for optimization involved only PMPA. Therefore, solvents in which the benzylation reaction was tested included solvents commonly used for GC–MS analysis such as dichloromethane (DCM, bp: 40 °C), acetonitrile (bp: 82 °C), acetone (bp: 56 °C) and ethyl acetate (EtOAc, bp: 77 °C). The benzylation of PMPA was observed to proceed more efficiently when acetonitrile and acetone were used as solvents in contrast to DCM and EtOAc where the yield of the benzyl esters was found to be clearly lower mirroring previously published results^27^ (Fig. 2c). Nevertheless, significant formation of the product was still observed in these other solvents. From these data, it was decided to conduct the reaction in acetonitrile during the last set of parameter optimization reactions that involved the absence and use of the acid additive and base as well. The benzylation of PMPA was found to work smoothly under basic conditions yielding BPMPA in comparable amounts to those obtained with added catalytic acid (5 mol% to trichloroacetimidate) as well as where no additive was part of the equation (Fig. 2d). Conditions where no additive was placed in the mixture were referred to as neutral, however even though these do not involve the presence of an acid or base, the mixture is acidic in nature due to the sole presence of the phosphonic acid itself. Consequently, it can be envisioned that the phosphonic acid can act as the catalyst for the benzylation outside any acid additive (Fig. 2e). Ultimately, the protocol yields three benzylated products that can be found in the instrument’s internal NIST library as well as the OCAD library and these are, along with their retention times, benzyl ethyl methylphosphonic acid BEMPA (t = 19.15 min.), benzyl pinacolyl methylphosphonic acid BPMPA (t = 22.42 and 22.69 min.) and benzyl cyclohexyl methylphosphonic acid BCMPA (t = 25.26 min.)

**Figure 3:**
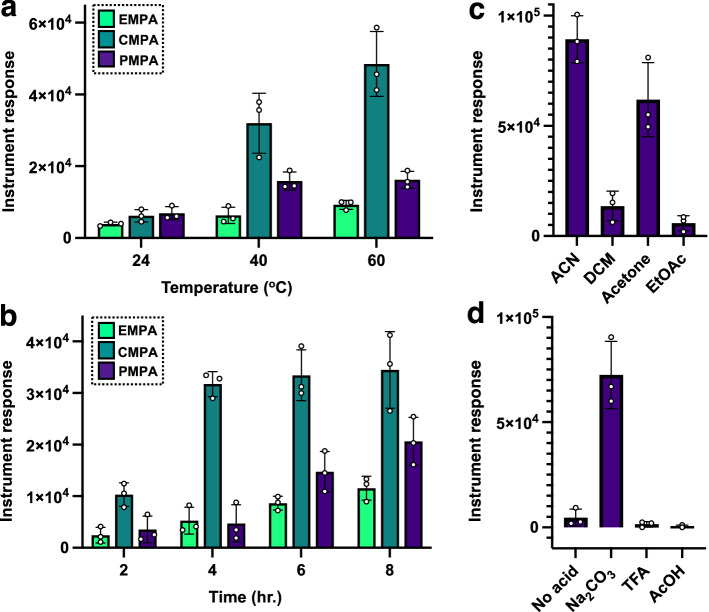
Derivatization of phosphonic acids with *p*-methoxybenzyl trichloroacetimidate: Optimization reactions, average (n = 3) peak areas (± the standard deviation) for the *p*-methoxybenzylated phosphonic acid after the acid (0.8 μmol, [100 ppm]) is treated with the trichloroacetimidate (200 μL, excess) in acetonitrile (ACN, 1 mL), (**a**) the effect of temperature on the derivatization yielding similar results for mild heating (40 °C) and heating to 60 °C for 4 h; (**b**) time for the reaction where a maximum accumulation of the three *p*-methoxybenzylated acids can be observed at ~ 4 h; (**c**) studies on just PMPA reveal that ACN and acetone are again optimal solvents for the derivatization; and (**d**) effect of no additives (‘neutral’), base (20 mg, excess) and catalytic acid (TFA and AcOH, 0.08 μmol (10 mol% relative to phosphonic acid) on the reaction demonstrates that the reaction works well only under basic conditions due presumably to the larger acid-sensitive nature of the PMB group.

Another trichloroacetimidate included in our studies was *p*-methoxybenzyl trichloroacetimidate. Reaction between *p*-methoxybenzyl trichloroacetimidate and the phosphonic acids occurs via the same mechanism as the one described for benzyl trichloroacetimidate (Fig. 2e), but the resulting phosphonic acid ester is composed of a benzyl unit that bears a methoxy substituent in its aromatic ring. The reason behind exploring this other trichloroacetimidate is the potential use of the *p*-methoxybenzyl (PMB) moiety to enhance the detection of the phosphonic acid relative to the unsubstituted benzyl counterpart by virtue of its electron-capture capabilities^33^. However, it is this ring substitution that also makes the *p*-methoxybenzyl group labile to mild acid^43,44^, thus necessitating the use of a base for the reaction to show some degree of success. The three products that arise from this specific derivatization along with their retention times in parentheses are *p*-methoxybenzyl ethyl methylphosphonic acid PMB-EMPA (t = 22.77 min.), *p*-methoxybenzyl pinacolyl methylphosphonic acid PMB-PMPA (t = 25.57 and 25.85 min.) and *p*-methoxybenzyl cyclohexyl methylphosphonic acid PMB-CMPA (t = 28.23 min.). It can be observed from the results on Fig. 3a–c, that the installation of the *p*-methoxybenzyl group onto the phosphonic acids as a function of temperature, time and solvent medium is very similar to the one displayed by its benzyl trichloroacetimidate counterpart (Fig. 2) under basic conditions. Thus, the *p*-methoxybenzylation appears to provide significant yield of the benzylated phosphonic acids at 40 °C and after 4 h (Fig. 3a,b) with slight yield improvement with increased temperature (i.e., 60 °C) or prolonged reaction time (up to 8 h). Regarding the reaction’s performance in different solvents, once again it was found that acetonitrile and acetone were the most optimal mediums for the derivatization to take place. Lastly, the acid lability of the *p*-methoxybenzyl group relative to that of its benzyl counterpart has a detrimental effect in the reaction’s yield as appreciated in Fig. 3d. Thus, it can be inferred from the results in Fig. 3d that even the catalytic use of acid does not aid in the esterification of PMPA as the medium is already acidic in nature and although some *p*-methoxybenzyl PMPA can be detected, this amount is minimal in comparison where a base (Na_2_CO_3_) is added in excess to counteract the hydrolytic effects of the acid (Fig. 3d).

**Figure 4:**
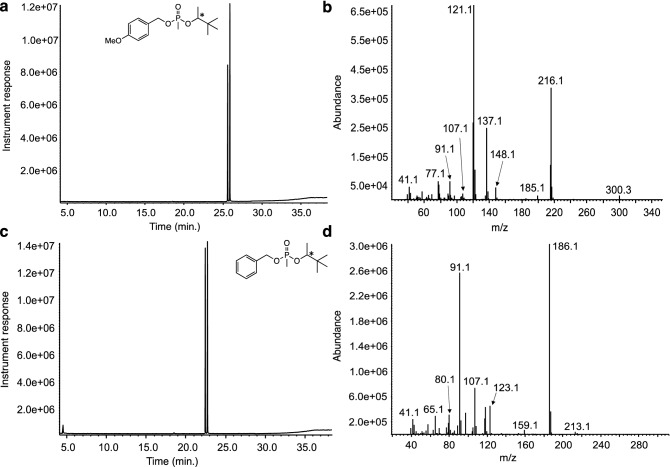
GC–MS traces and associated mass spectra for PMB-PMPA and BPMPA. The GC–MS data was gathered on synthesized standards. Note the resolution of both diastereomers of PMPA by the column as a result of the two stereogenic centers in the molecule, the P atom and the C atom in the pinacolyl arm marked by an asterisk (*).

Mass spectral comparison of both PMPA derivatives show very interesting characteristics that reflect their unique structural make up that greatly aid the analyst in the analysis of this important nerve agent degradation marker (Fig. 4). Starting with the retention times associated with both benzylated PMPA compounds, one can observe that these are vastly different with retention times of 25.57 and 25.85 min for PMB-PMPA (Fig. 4a) while the retention times for BPMPA were found to be 22.42 and 22.69 min. As expected, the GC–MS profile for derivatives of PMPA appear as two signals arising from the diastereomeric mixture of the acids^28,29^. Analysis of the mass spectrum for PMB-PMPA shows two key features, the first one is that a molecular ion peak (m/z = 300.3) is clearly visible and that the base peak is m/z = 121.1 assigned the methoxy-substituted tropylium cation (Fig. 4b). Other peaks of interest in the spectrum of PMB-PMPA include the peak at m/z = 216.1 which is a fragment that arises from the loss of the alkyl side chain of the pinacolyl moiety to yield a PMB-substituted phosphonic acid (C_9_H_13_O_4_P) and m/z = 137.1 belonging to the PMB-O side unit of PMB-PMPA with a tentative formula of C_8_H_9_O_2_ (Fig. 4b). In contrast, the mass spectrum for BPMPA shows few peaks but two of these are very intense (Fig. 4d). The first one is the base peak (m/z = 186.1) which is a fragment that arises from the loss of the alkyl side chain of the pinacolyl moiety to yield a benzyl-substituted phosphonic acid (C_8_H_11_O_3_P) and m/z = 91.1 which indicates the formation of the tropylium ion. As expected during EI-GC–MS analyses, the molecular ion peak for some derivatives may not be observed and this is the case for BPMPA (m/z = 270) (Fig. 4d).

### Benzylation of pinacolyl methylphosphonic acid in OPCW-PT soil sample

Pinacolyl methylphosphonic acid represents the first degradation product of the nerve agent Soman (GD) and depending on the environmental or biological conditions where it is found, it could be the most abundant species arising from this nerve agent in addition to methylphosphonic acid. Soman is a nerve agent belonging to the G-series that possesses the shortest aging time once it is bound to the enzyme acetylcholinesterase (AChE) and as such it has become one of the most lethal ones among the already very toxic nerve agents^45^. Aside from this lethal characteristic, GD and PMPA in derivatized form have a unique behavior during GC–MS analyses and is that two signals are always observed for these analytes as they exist as a diastereomeric mixture^28,29^. This is a unique characteristic as GD is the only nerve agent from the G-series that possesses two chiral centers (at C and P centers) and as such it has become a constantly featured analyte during OPCW proficiency tests (PTs). The main goal during an OPCW-PT is the eventual detection and identification of chemical warfare agent related degradation products. During these PTs, it is important not only to detect but to unambiguously identify an analyte by comparing its chromatographic retention time and its mass spectrum to those of a known reference chemical whether it is on its native or derivatized form^46^.

**Figure 5:**
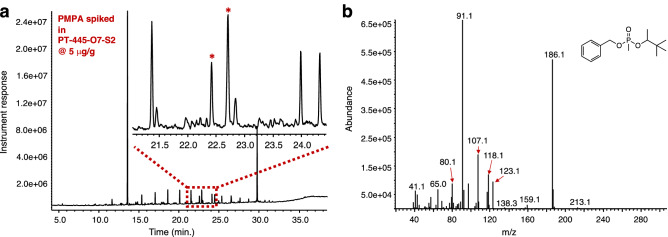
GC–MS analysis on soil sample PT-S2* containing PMPA at ~ 5 μg/g concentration. (**a**) Gas chromatogram showing the formation of the benzyl product BPMPA (indicated by both asterisks); (**b**) mass spectrum of BPMPA.

Thus, after demonstrating the ability of the method to convert phosphonic acids into their corresponding benzyl esters we turned our attention to testing its performance on a soil sample featured in the 44th OPCW-PT^35^. The PT soil sample, designated with the code PT-S2, contained within its matrix the reportable compound pinacolyl methylphosphonic acid (PMPA, Schedule 2.B.04) that was found afterwards to be spiked at a concentration of 50 μg/g. The soil sample was combined with the soil matrix that is given to the participating laboratory as a blank sample to generate a diluted sample called PT-S2* that now contained the PMPA at a 5 μg/g concentration. The soil sample PT-S2, aside from containing organic matter detectable by GC–MS, was also spiked with a series of homologous hydrocarbons presumably to act as interfering species during the PT analyses. The protocol consisted in taking up soil matrix PT-S2* in ACN (2 mL) and treating the suspension simultaneously with Na_2_CO_3_ (20 mg) and benzyl 2,2,2-trichloroacetimidate (40 μL). The resulting mixture was heated in a heat block for 4 h, filtered, the ACN filtrate concentrated with a nitrogen stream to ~ 150 μL and analyzed by GC–MS. As it can be seen in Fig. 5a, the formation of BPMPA using the optimized protocol can be observed when PMPA is in such low concentration (5 μg/g). Careful analysis of the mass spectrum arising from either one of the peaks protruding among the various soil interferences shows that indeed, these peaks arise from the benzylated PMPA product (Fig. 5b). The method’s MDL values for the benzyl and the *p*-methoxybenzyl pinacolyl methylphosphonic acids for this matrix were determined to be 35 and 63 ng/mL respectively following the established guidelines by EPA (EPA-821-R16-006). In addition, using the same guidelines, the LOQ for the method for both, the benzyl and the *p*-methoxybenzyl pinacolyl methylphosphonic acids were determined to be 104 and 189 ng/mL respectively (Table 1).

**Table 1:** MDL and LOQ values calculated for each derivatized PMPA analyte in the OPCW-PT soil sample.

	BPMPA (QI-m/z 91)	PMB-PMPA (QI-m/z 121)
MDL (ng/mL)	35	63
LOQ (ng/mL)	104	189

QI are the quantitation ions used in the calculation for each analyte.

### Benzylation of pinacolyl methylphosphonic acid in OPCW-PT fatty acid-rich matrix

**Figure 6:**
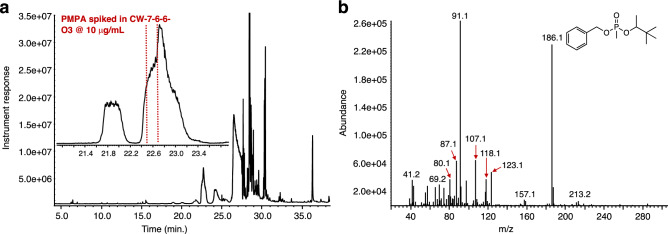
GC–MS analysis on fatty acid-rich matrix CW-O3 spiked with PMPA at ~ 10 μg/mL concentration. (**a**) GC chromatogram of CW-7-6-6-03 matrix after reaction with benzyl 2,2,2-trichloroacetimidate. A clear signal of the product BPMPA cannot be observed due to numerous interferences in the matrix, even after extracting selected ions (m/z = 91, 186; inset). (**b**) Mass analysis of the area indicated by the red dotted lines in Fig. 5a inset yields the mass spectrum for BPMPA.

The method was tested for its efficiency at benzylating PMPA in a fatty acid-rich liquid matrix featured in the 38th OPCW-PT^28^. The liquid sample featured during this PT (code: CW-O3) administered in 2015 consisted of a number of scheduled chemicals that included pinacolyl alcohol^47,48^ the other end hydrolysis product of PMPA aside from methylphosphonic acid spiked in a fatty acid-rich matrix that made their analysis and identification extremely difficult. Derivatization approaches that included silylation and methylation were carried out with limited success forcing efforts that included a prior purification of the matrix via column chromatography using silica gel and hexanes as the eluting solvent. For our studies herein, PMPA was spiked in this matrix at a ~ 10 μg/mL concentration. Treatment of the spiked matrix with excess benzyl trichloroacetimidate at 60 °C for 4 h resulted in the formation of BPMPA (Fig. 6). Interestingly, the NIST internal library in our instrument could not provide a clear match to BPMPA, matching its mass spectrum to that of *n*-butylbenzylphosphonic acid (BBPA), a very similar analog (Match score to BBPA = 759 (49%)). This is in stark contrast to its PMB-PMPA counterpart for which no matching compounds were obtained when using the instrument’s internal library search^39^.

## Experimental section

### Chemicals and reagents

All chemicals were purchased from commercial suppliers and used as received, ethyl methylphosphonic acid (EMPA), cyclohexyl methylphosphonic acid (CMPA), pinacolyl methylphosphonic acid (PMPA), benzyl 2,2,2-trichloroacetimidate, 4-methoxybenzyl-2,2,2-trichloroacetimidate, acetonitrile (ACN), acetone, ethyl acetate and dichloromethane were purchased from Sigma-Aldrich (St. Louis, MO.). Sodium bicarbonate, sodium carbonate and anhydrous sodium sulfate were purchased from Acros Organics (Westchester, PA). Deuterated chloroform (CDCl_3_) was purchased from Alfa Aesar (Ward Hill, MA). Acrodisc PTFE syringe filters (0.45 μm) were purchased from Pall laboratories (Port Washington, NY.). Autosampler vials and glass inserts were purchased from Agilent Technologies (Santa Clara, CA.). Soil sample PT-S2 and liquid matrix CW-O3 were obtained from the Forensic Science Center (FSC) OPCW-PT sample archives at the Lawrence Livermore National Laboratory (LLNL).

### Spiking of PMPA in OPCW-PT soil and liquid samples for testing

A stock solution of PMPA (1 mg/mL, 1000 ppm) was prepared in DCM (10 mL) and this solution was used for the spiking of the soil sample (code: PT-S2) and the fatty acid-rich liquid sample (code: CW-O3). The stock solution was kept in the refrigerator (4 °C) and only taken out when used for dilutions or spiking of the matrices. The soil sample was prepared as follows, soil PT-S2 (200 mg) was treated with PMPA (10 μL of stock solution, 0.01 mg) and the mixture made into a suspension in DCM (1 mL) and vortexed to assure a uniformly spiked soil sample. The DCM was evaporated with a gentle nitrogen stream to give a soil sample containing PMPA in a 5 μg/g concentration. The liquid sample was prepared as follows, liquid CW-O3 (200 μL) was diluted with DCM (200 μL) and treated with PMPA (40 μL of stock solution, 0.04 mg) to provide a liquid matrix spiked with PMPA at a 10 μg/mL concentration.

### GC–MS analysis

A 6890 Agilent GC with a 5975 MS detector equipped with a split/splitless injector was used for the analysis in the splitless mode. The GC column used for the analysis was an Agilent DB-5MS capillary column (30 m × 0.25 mm id × 0.25 μm film thickness). Ultra-high purity helium was used as the carrier gas at 0.8 mL/min. The injector temperature was 250 °C, and the injection volume was 1 μL. The oven temperature program was as follows: 40 °C, held for 3 min, increased at 8 °C/min to 300 °C, held for 3 min. The MS ion source and quadrupole temperatures were 230 and 150 °C, respectively. Electron ionization was used with ionization energy of 70 eV. The MS was operated to scan from *m/z* 29 to *m/z* 600 in 0.4 s. with a solvent delay of 3.5 min. as described previously^28^.

### Nuclear magnetic resonance analysis

Spectra were obtained using a Bruker Avance III 600 MHz instrument equipped with a Bruker TCI 5 mm cryoprobe (Bruker Biospin, Billerica, MA) at 30.0 ± 0.1 °C. ^1^H NMR (600 MHz), ^13^C/^13^C (DEPT-135) NMR (150 MHz) and ^31^P/^31^P{^1^H} NMR were recorded in CDCl_3_. NMR data is reported as follows: chemical shift (δ) (parts per million, ppm); multiplicity: d (doublet), app ten (apparent tentet), qd (quartet of doublets); coupling constants (*J*) are given in Hertz (Hz). ^1^H NMR chemical shifts are calibrated with respect to the residual CHCl_3_ singlet centered at 7.26 ppm while for ^13^C NMR the triplet centered at 77.16 ppm from CDCl_3_ was used for the spectral calibration.

### Chemical synthesis

Solvents used during the syntheses were removed by using a Büchi rotary evaporator R-200 equipped with a Büchi heating bath B-490 and coupled to a KNF Laboport Neuberger UN820 vacuum pump. Analytical thin layer chromatography (TLC) was conducted on Agela Technologies silica gel glass plates coupled with detection by ceric ammonium molybdate (CAM)^49–51^, exposure to iodine vapor and/or UV light (λ = 254 nm)^52–55^. Purification of the benzylated standards was accomplished using a Biotage Purification System using silica gel cartridges coupled to a UV detection system (λ = 245, 365 nm). HRMS analyses were obtained at the Forensic Science Center at the Lawrence Livermore National Laboratory using Chemical Ionization (CI). *Benzyl pinacolyl methylphosphonate*: Pinacolyl methylphosphonic acid (150 mg, 0.83 mmol) was dissolved in DMF (5 mL) and treated sequentially with sodium carbonate (160 mg, 1.51 mmol, 1.83 equiv. to acid) and benzyl 2,2,2-trichloroacetimidate (183 μL, 0.99 mmol, 1.2 equiv.). After the overnight stirring, water (5 mL) was added to the scintillation vial and the organic phase extracted, dried over anhydrous sodium sulfate, passed through a syringe filter disk, and evaporated *in vacuo* to give a yellow oil that was purified by flash column chromatography (hexanes → 7:3 EtOAc/hexanes) to give benzyl pinacolyl methylphosphonate as a colorless oil (184 mg, 82%). ^1^H NMR (diastereomeric mixture: 1.5:1 (maj = *major*; min = *minor*, 600 MHz, CDCl_3_) δ 5.11 (dd, *J* = 11.9, 8.0 Hz, 1H^maj^), 5.06 (dd, *J* = 11.9, 8.1 Hz, 1H^min^), 5.04 (dd, *J* = 16.9, 8.4 Hz, 1H^maj^), 5.01 (dd, *J* = 17.0, 8.3 Hz, 1H^min^), 4.26 (dq, *J* = 8.3, 6.4 Hz, 1H^min^), 4.19 (dq, *J* = 8.3, 6.4 Hz, 1H^maj^), 1.46 (d, *J* = 3.9 Hz, 3H^min^), 1.43 (d, *J* = 3.8 Hz, 3H^maj^), 1.26 (d, *J* = 6.3 Hz, 3H^maj^), 1.23 (d, *J* = 6.3 Hz, 3H^min^), 0.90 (s, 9H^min^), 0.89 (s, 9H^maj^); ^13^C NMR (150 MHz, CDCl_3_) δ 136.57, 136.54, 136.50, 128.48, 128.19, 127.68, 127.67, 81.36, 81.30, 81.14, 81.09, 66.94, 66.90, 66.71, 66.68, 34.89, 34.87, 34.85, 34.83, 25.52, 25.51, 17.03, 16.86, 12.99, 12.14, 12.02, 11.17; ^31^P{^1^H} NMR (242 MHz, CDCl_3_) δ 30.50, 29.81; ^31^P NMR δ 30.49 (app ten, *J* = 8.4 Hz, P^min^), 29.80 (app ten, *J* = 8.4 Hz, P^maj^); HRMS (CI) *m/z* calcd for C_14_H_23_O_3_P [M^+^]: 270.1385; found 270.1401. *p*-Methoxybenzyl pinacolyl methylphosphonate: The reaction was similar to the one for the benzyl counterpart with the only difference lying in the use of *p*-methoxybenzyl trichloroacetimidate (205 μL, 0.99 mmol). The *p*-methoxybenzyl pinacolyl methylphosphonate product was obtained as a colorless oil after purification by flash column chromatography (hexanes → 7:3 EtOAc/hexanes) (159 mg, 64%). ^1^H NMR (diastereomeric mixture, 600 MHz, CDCl_3_) δ 7.32 (d, *J* = 8.2 Hz, 2H), 6.89 (d, *J* = 8.2 Hz, 2H), 5.07–4.92 (m, 2H^maj,min^), 4.25 (dq, *J* = 8.3, 6.3 Hz, 1H^maj^), 4.18 (dq, *J* = 8.3, 6.3 Hz, 1H^min^), 3.80 (s, OCH_3_, 3H^maj,min^), 1.44 (d, *J* = 4.4 Hz, 3H^maj^), 1.41 (d, *J* = 4.3 Hz, 3H^min^), 1.27 (d, *J* = 6.4 Hz, 3H^min^), 1.24 (d, *J* = 6.4 Hz, 3H^maj^), 0.91 (s, 9H^maj^), 0.89 (s, 9H^min^); ^13^C NMR (150 MHz, CDCl_3_) δ 159.67, 130.02, 129.64, 129.62, 128.84, 128.80, 128.74, 128.70, 113.93, 81.24, 81.19, 81.03, 80.98, 66.86, 66.81, 66.64, 66.60, 55.27, 34.93, 34.91, 34.89, 34.87, 25.58, 17.07, 16.90, 13.14, 12.30; ^31^P{^1^H} NMR (242 MHz, CDCl_3_) δ 30.37, 29.66; ^31^P NMR δ 30.36 (app ten, *J* = 8.3 Hz, P^maj^), 29.66 (app ten, *J* = 8.3 Hz, P^min^); HRMS (CI) *m/z* calcd for C_15_H_25_O_4_P [M^.+^]: 300.1490; found 300.1497. Spectral data for these two benzylated PMPA standards is provided in the Supporting Information.

## Conclusion

The use of benzyl- and *p*-methoxybenzyl trichloroacetimidate for the derivatization of phosphonic acid nerve agent markers under neutral, basic and slightly acidic conditions has been evaluated. The phosphonic acids used included ethyl-, cyclohexyl- and pinacolyl methylphosphonic acid, that represent signature hydrolysis products from the nerve agents ethyl *N*-2-diisopropylaminoethyl methylphosphonothiolate (VX), cyclosarin (GF) and soman (GD) respectively. The optimized conditions for the derivatization of the phosphonic acids included neutral, basic as well as catalytic acid (< 5 mol%) with the trichloroacetimidate in excess coupled to heating the mixture at 60 °C in acetonitrile for 4 h. Comparative studies demonstrated that while the benzylation of the phosphonic acids proceeded smoothly under all three conditions (neutral, acid and basic), the *p*-methoxybenzylation of these was found to work efficiently only in the presence of a base (e.g., Na_2_CO_3_). This in agreement with the lability of the *p*-methoxy benzyl group under mild to highly acidic conditions. The method’s efficiency was tested in the successful derivatization and identification of pinacolyl methylphosphonic acid (PMPA) as its benzyl ester when present at a concentration of ~ 5 μg/g in a soil sample featured in the 44th OPCW-PT. In addition, the protocol was used in the detection and identification of PMPA when spiked at ~ 10 μg/mL concentration in a fatty acid-rich liquid matrix featured in the 38th OPCW-PT. The benzyl derivative of PMPA was corroborated with the instrument's internal NIST spectral library or the OPCW central analytical database (OCAD v.21_2019) as well as authentic standards synthesized in our laboratory. The MDL values calculated for the benzyl and the *p*-methoxybenzyl pinacolyl methylphosphonic acids were determined to be 35 and 63 ng/g respectively in the soil extract featured in the 44th OPCW-PT (Supplementary Information).

## Supplementary Information

Supplementary Information.

## Data Availability

All data generated or analysed during this study are included in this published article and its supplementary information files.

## References

[1] Chai PR, Boyer EW, Al-Nahhas H, Erickson TB (2007). Toxic chemical weapons of assassination and warfare: Nerve agents VX and sarin. Toxicol. Commun..

[2] Stone R (2018). UK attack puts nerve agent in the spotlight. Science.

[3] Stone R (2020). How German military scientists likely identified the nerve agent used to attack Alexei Navalny. Science.

[4] Thiermann H, Worek F, Kehe K (2013). Limitations and challenges in treatment of acute chemical warfare agent poisoning. Chem. Biol. Interact..

[5] Worek F, Thiermann H, Wille T (2016). Oximes in organophosphate poisoning: 60 years of hope and despair. Chem. Biol. Interact..

[6] Bennion BJ (2021). Development of a CNS-permeable reactivator for nerve agent exposure: An iterative, multi-disciplinary approach. Sci. Rep..

[7] Kim K, Tsay OG, Atwood DA, Churchill DG (2011). Destruction and detection of chemical warfare agents. Chem. Rev..

[8] Mayer BP, Valdez CA, Hok S, Chinn SC, Hart BR (2012). ^31^P-edited diffusion-ordered ^1^H NMR spectroscopy for the spectral isolation and identification of organophosphorus compounds related to chemical weapons agents and their degradation products. Anal. Chem..

[9] Black RM, Clarke RJ, Read RW, Reid MTJ (1994). Application of gas chromatography-mass spectrometry and gas chromatography-tandem mass spectrometry to the analysis of chemical warfare samples, found to contain residues of the nerve agent sarin, sulphur mustard and their degradation products. J. Chromatogr. A..

[10] D’Agostino PA, Hancock JR, Chenier CL (2003). Mass spectrometric analysis of chemical warfare agents and their degradation products in soil and synthetic samples. Eur. J. Mass Spectrom..

[11] Tu AT (2014). Aum Shinrikyo's chemical and biological weapons: More than sarin. Forensic Sci. Rev..

[12] Okumura T (2005). The Tokyo subway sarin attack—Lessons learned. Toxicol. Appl. Pharmacol..

[13] Dolgin E (2013). Syrian gas attack reinforces need for better anti-sarin drugs. Nat. Med..

[14] Butler D (2018). Attacks in UK and Syria highlight growing need for chemical-forensics expertise. Nature.

[15] Tu A (2020). The use of VX as a terrorist agent: Action by Aum Shinrikyo of Japan and the death of Kim Jong-Nam in Malaysia: four case studies. Glob. Security Health Sci. Policy..

[16] Valdez CA, Leif RN (2021). Analysis of organophosphorus-based nerve agent degradation products by gas chromatography-mass spectrometry (GC-MS): Current derivatization reactions in the analytical chemist’s toolbox. Molecules.

[17] Dawson RM, Pantelidis S, Rose HR, Kotsonis SE (2008). Degradation of nerve agents by an organophosphate degrading agent (OpdA). J. Hazard Mater..

[18] Yang Y-C (1999). Chemical detoxification of nerve agent VX. Acc. Chem. Res..

[19] Valdez CA, Leif RN, Hok S, Hart BR (2018). Analysis of chemical warfare agents by gas chromatography-mass spectrometry: Methods for their direct detection and derivatization approaches for the analysis of their degradation products. Rev. Anal. Chem..

[20] Black RM, Muir B (2003). Derivatisation reactions in the chromatography analysis of chemical warfare agents and their degradation products. J. Chromatogr. A.

[21] Jansson D (2018). Part 2: Forensic attribution profiling of Russian VX in food using liquid chromatography-mass spectrometry. Talanta.

[22] Holmgren KH (2018). Part 1: Tracing Russian VX to its synthetic routes by multivariate statistics of chemical attribution signatures. Talanta.

[23] Williams AM, Vu AK, Mayer BP, Hok S, Valdez CA, Alcaraz A (2018). Part 3: Solid phase extraction of Russian VX and its chemical attribution signatures in food matrices and their detection by GC-MS and LC-MS. Talanta.

[24] Ovenden SPB (2020). The identification of chemical attribution signatures of stored VX nerve agents using NMR, GC-MS, and LC-HRMS. Talanta.

[25] Webster RL (2022). Chemical forensic profiling and attribution signature determination of sarin nerve agent using GC-MS, LC-MS and NMR. Anal. Bioanal. Chem..

[26] Richardson DD, Caruso JA (2007). Derivatization of organophosphorus nerve agent degradation products for gas chromatography with ICPMS and TOF-MS detection. Anal. Bioanal. Chem..

[27] Motojyuku M (2008). Determination of glyphosate, glyphosate metabolites, and glufosinate in human serum by gas chromatography-mass spectrometry. J. Chromatogr. B Anal. Technol. Biomed. Life Sci..

[28] Valdez CA, Leif RN, Alcaraz A (2016). Effective methylation and identification of phosphonic acids relevant to chemical warfare agents mediated by trimethyloxonium tetrafluoroborate for their qualitative detection by gas chromatography-mass spectrometry. Anal. Chim. Acta..

[29] Valdez CA, Marchioretto MK, Leif RN, Hok S (2018). Efficient derivatization of methylphosphonic and aminoethylsulfonic acids related to nerve agents simultaneously in soils using trimethyloxonium tetrafluoroborate for their enhanced, qualitative detection and identification by EI-GC-MS and GC-FPD. Forensic Sci. Int..

[30] Macomber RS (1977). Esterification of phosphonic acids with diazomethane. Synth. Commun..

[31] Amphaisri K, Palit M, Mallard G (2011). Thermally assisted methylation and subsequent silylation of scheduled acids of chemical weapon convention for on- site analysis and its comparison with the other methods of methylation. J. Chromatogr. A..

[32] Halket J, Zaikin V (2004). Derivatization in mass spectrometry—3. alkylation (arylation). Eur. J. Mass Spectrom..

[33] Palit M, Gupta AK, Jain R, Raza SK (2004). Determination of pentafluorobenzyl derivatives of phosphonic and phosphonothioic acids by gas chromatography-mass spectrometry. J. Chromatogr. A..

[34] Knapp DR (1979). Handbook of Analytical Derivatization Reactions.

[35] Valdez CA, Leif RN, Hok S, Vu AK, Salazar EP, Alcaraz A (2019). Methylation protocol for the retrospective detection of isopropyl-, pinacolyl- and cyclohexylmethylphosphonic acids, indicative markers for the nerve agents sarin, soman and cyclosarin, at low levels in soils using EI-GC-MS. Sci. Total Environ..

[36] van’t Erve TJ, Rautiainen RH, Robertson LW, Luthe G (2010). Trimethylsilyldiazomethane: A safe non-explosive, cost effective and less-toxic reagent for phenol derivatization in GC applications. Environ. Int..

[37] Pagliano E (2020). Versatile derivatization for GC-MS and LC-MS: Alkylation with trialkyloxonium tetrafluoroborates for inorganic anions, chemical warfare agent degradation products, organic acids, and proteomic analysis. Anal. Bioanal. Chem..

[38] Standard Reference Data Program, National Institute of Standards and Technology, Gaithersburg, MD. Standard reference database IA (2011) http://www.nist.gov/srd/nist1a.htm.

[39] Valdez CA, Leif RN, Hok S, Alcaraz A (2018). Assessing the reliability of the NIST library during routine GC-MS analyses: Structure and spectral data corroboration for 5,5-diphenyl-1,3-dioxolan-4-one during a recent OPCW proficiency test. J. Mass Spectrom..

[40] Nyanyira, C. *The OPCW Central Analytical Database, Chemical Weapons Convention Chemicals Analysis: Sample Collection, Preparation and Analytical Methods* (ed Mesilaakso, M.) 133–149 (Wiley, 2005).

[41] Otsuka M, Tsuge K, Seto Y, Miyaguchi H, Uchiyama M (2018). Analysis of degradation products of nerve agents via post-pentafluorobenzylation liquid chromatography-tandem mass spectrometry. J. Chromatogr. A..

[42] Hamachi A, Imasaka T, Nakamura H, Li A, Imasaka T (2017). Determination of nerve agent metabolites by ultraviolet femtosecond laser ionization mass spectrometry. Anal. Chem..

[43] Iversen T, Bundle DR (1981). Benzyl trichloroacetimidate, a versatile reagent for acid-catalysed benzylation of hydroxy-groups. J. Chem. Soc. Chem. Commun..

[44] Eckenberg P, Groth U, Huhn T, Richter N, Schmeck C (1993). A useful application of benzyl trichloroacetimidate for the benzylation of alcohols. Tetrahedron.

[45] Stone R (2018). How to defeat a nerve agent. Science.

[46] Blum M-M, Murty MRVS (2014). Analytical chemistry and the chemical weapons convention. Anal. Bioanal. Chem..

[47] Valdez CA (2021). Acylation as a successful derivatization strategy for the analysis of pinacolyl alcohol in a glycerol-rich matrix by GC-MS: Application during an OPCW Proficiency Test. Anal. Bioanal. Chem..

[48] Albo RLF, Valdez CA, Leif RN, Mulcahy HA, Koester C (2014). Derivatization of pinacolyl alcohol with phenyldimethylchlorosilane for enhanced detection by gas chromatography-mass spectrometry. Anal. Bioanal. Chem..

[49] Wnuk SF (2000). Doubly homologated dihalovinyl and acetylene analogues of adenosine. Synthesis, interaction with S-adenosyl-l-homocysteine hydrolase, and antiviral and cytostatic effects. J. Med. Chem..

[50] Wnuk SF (2002). Sugar-modified conjugated diene analogues of adenosine and uridine. Synthesis, interaction with S-adenosyl-l-homocysteine hydrolase, and antiviral and cytostatic effects. J. Med. Chem..

[51] Showalter BM (2005). Diazeniumdiolate ions as leaving groups in anomeric displacement reactions: A protection-deprotection strategy for ionic diazeniumdiolates. J. Am. Chem. Soc..

[52] Valdez CA (2009). Synthesis and electrochemistry of 2-ethenyl and 2-ethanyl derivatives of 5-nitroimidazole and antimicrobial activity against Giardia lamblia. J. Med. Chem..

[53] Abramson D, Blecher M (1964). Quantitative two-dimensional thin-layer chromatography of naturally occurring phospholipids. J. Lipid. Res..

[54] Barrett GC (1962). Iodine as a ‘non-destructive’ colour reagent in paper and thin-layer chromatography. Nature.

[55] Valdez CA (2008). Hydrolytic reactivity trends among potential prodrugs of the O_2_-glycosylated diazeniumdiolate family. Targeting nitric oxide to macrophages for antileishmanial activity. J. Med. Chem..

