# Supplementary material for: Benzyl trichloroacetimidates as derivatizing agents for phosphonic acids related to nerve agents by EI-GC-MS during OPCW proficiency test scenarios
Source: Sci Rep. 2022 Dec 9;12:21299. doi: 10.1038/s41598-022-25710-4 (PMC9734645; doi:10.1038/s41598-022-25710-4)
Supplement: Supplementary file 1 — Supplementary Information. [file 41598_2022_25710_MOESM1_ESM.docx]

**- Supporting Information -**

**Benzyl Trichloroacetimidates as Derivatizing Agents for Phosphonic Acids Related to Nerve Agents by EI-GC-MS During OPCW Proficiency Test Scenarios**

Alagu Subramanian^1-3^, José A. Rosales^1,4,5^, Roald N. Leif^1,2,5^, Carlos A. Valdez^1,2,5,^*

^1^Forensic Science Center, ^2^Nuclear and Chemical Sciences Division, ^3^Biosciences and Biotechnology Division, ^4^NNSA-MSIIP Summer Fellow, University of Texas, El Paso, TX, USA. ^5^Global Security Directorate, Lawrence Livermore National Laboratory, Livermore, CA, USA.

**Table of Contents**

**Content Page**

Optimization data for benzylation of phosphonic acids 2

Optimization data for *p*-methoxybenzylation of phosphonic acids 3

NMR spectra acquisition 4

^1^H NMR spectrum of BPMPA 4

^13^C NMR spectrum of BPMPA 5

^13^C DEPT-135 NMR spectrum of BPMPA 5

^31^P NMR spectrum of BPMPA 6

^31^P{^1^H} NMR spectrum of BPMPA 6

^1^H NMR spectrum of PMB-PMPA 7

^13^C NMR spectrum of PMB-PMPA 7

^13^C DEPT-135 NMR spectrum of PMB-PMPA 8

^31^P NMR spectrum of PMB-PMPA 8

^31^P{^1^H} NMR spectrum of PMB-PMPA 9

MDL calculations data 10

**Optimization studies for the benzylation reaction of phosphonic acids**

| EMPA | | | CMPA | | | PMPA | | |
| --- | --- | --- | --- | --- | --- | --- | --- | --- |
| 24 | 40 | 60 | 24 | 40 | 60 | 24 | 40 | 60 |
| 3233 | 6899 | 5653 | 6090 | 24556 | 35778 | 4990 | 8909 | 11012 |
| 4890 | 4588 | 4467 | 9034 | 28966 | 32455 | 6023 | 8823 | 15992 |
| 3122 | 6865 | 6667 | 5465 | 34212 | 41421 | 7809 | 6022 | 16992 |

**Table S1.** Effect of temperature on benzylation of phosphonic acids (EMPA, CMPA and PMPA). Areas under the curves for all benzylated acids. For PMPA, the summation of both diastereomeric peaks is given.

| EMPA | | | | CMPA | | | | PMPA | | | |
| --- | --- | --- | --- | --- | --- | --- | --- | --- | --- | --- | --- |
| 2 | 4 | 6 | 8 | 2 | 4 | 6 | 8 | 2 | 4 | 6 | 8 |
| 13423 | 30876 | 41232 | 45422 | 46787 | 76567 | 102345 | 113232 | 15678 | 34356 | 28654 | 35456 |
| 14567 | 25643 | 32343 | 32121 | 51232 | 66890 | 90134 | 94532 | 14543 | 38766 | 35898 | 29093 |
| 22122 | 24543 | 38987 | 33434 | 38676 | 75221 | 85612 | 72678 | 19233 | 21223 | 37809 | 35444 |

**Table S2.** Effect of time on benzylation of phosphonic acids (EMPA, CMPA and PMPA).

Areas under the curves for all benzylated acids. For PMPA, the summation of both diastereomeric peaks is given.

| PMPA | | | |
| --- | --- | --- | --- |
| ACN | DCM | Acetone | EtOAc |
| 59212 | 15668 | 44989 | 4812 |
| 66258 | 14787 | 45263 | 15284 |
| 70215 | 10989 | 60189 | 14301 |

**Table S3.** Effect of solvent on benzylation of PMPA.

| PMPA | | | |
| --- | --- | --- | --- |
| --- | Na_2_CO_3_ | TFA | AcOH |
| 80526 | 62536 | 89259 | 45815 |
| 77151 | 55818 | 57623 | 49256 |
| 89025 | 49632 | 61202 | 51878 |

**Table S4.** Effect on benzylation of PMPA when the reaction is run with no additives, sodium carbonate, trifluoroacetic acid (TFA) and acetic acid (AcOH).

**Optimization studies for the *p*-methoxybenzylation reaction of phosphonic acids**

| EMPA | | | CMPA | | | PMPA | | |
| --- | --- | --- | --- | --- | --- | --- | --- | --- |
| 24 | 40 | 60 | 24 | 40 | 60 | 24 | 40 | 60 |
| 4000 | 8909 | 10003 | 6090 | 22454 | 45654 | 5656 | 14533 | 15601 |
| 4243 | 4588 | 7787 | 8001 | 37877 | 58632 | 6454 | 18797 | 18777 |
| 2998 | 5421 | 9929 | 4545 | 35667 | 41232 | 9023 | 14323 | 14309 |

**Table S5.** Effect of temperature on benzylation of phosphonic acids (EMPA, CMPA and PMPA). Areas under the curves for all benzylated acids. For PMPA, the summation of both diastereomeric peaks is given.

| EMPA | | | | CMPA | | | | PMPA | | | |
| --- | --- | --- | --- | --- | --- | --- | --- | --- | --- | --- | --- |
| 2 | 4 | 6 | 8 | 2 | 4 | 6 | 8 | 2 | 4 | 6 | 8 |
| 4090 | 8234 | 9909 | 12344 | 10564 | 33421 | 39095 | 41232 | 6464 | 8787 | 10922 | 25411 |
| 2132 | 4122 | 7210 | 13331 | 12421 | 32778 | 31226 | 35654 | 1656 | 1898 | 14511 | 20312 |
| 1090 | 3423 | 8810 | 8934 | 7867 | 28976 | 29997 | 26545 | 2545 | 3454 | 18768 | 16098 |

**Table S6.** Effect of time on benzylation of phosphonic acids (EMPA, CMPA and PMPA).

Areas under the curves for all benzylated acids. For PMPA, the summation of both diastereomeric peaks is given.

| PMPA | | | |
| --- | --- | --- | --- |
| ACN | DCM | Acetone | EtOAc |
| 88202 | 15252 | 49528 | 1985 |
| 79251 | 6252 | 55123 | 6936 |
| 100484 | 19363 | 81024 | 8581 |

**Table S7.** Effect of solvent on benzylation of PMPA.

| PMPA | | | |
| --- | --- | --- | --- |
| --- | Na_2_CO_3_ | TFA | AcOH |
| 2633 | 90451 | -- | 1152 |
| 9251 | 66845 | 1958 | -- |
| 1858 | 59962 | 2414 | -- |

**Table S8.** Effect on benzylation of PMPA when the reaction is run with no additives, sodium carbonate, trifluoroacetic acid (TFA) and acetic acid (AcOH).

**NMR spectra acquisition**

Spectra were obtained using a Bruker Avance III 600 MHz instrument equipped with a Bruker TCI 5 mm cryoprobe (Bruker Biospin, Billerica, MA) at 30.0 ± 0.1 °C. ^1^H NMR (600 MHz), ^13^C/^13^C (DEPT-135) NMR (150 MHz) and ^31^P/^31^P{^1^H} NMR were recorded in CDCl_3_. ^1^H NMR chemical shifts are calibrated with respect to the residual CHCl_3_ singlet centered at 7.26 ppm while for ^13^C NMR the triplet centered at 77.16 ppm from CDCl_3_ was used for the spectral calibration.

**Figure S1**. ^1^H NMR of BPMPA.

**Figure S2.** ^13^C NMR of BPMPA.

**Figure S3.** ^13^C-DEPT-135 NMR of BPMPA.

**Figure S4.** ^31^P{^1^H} NMR of BPMPA.

**Figure S5.** ^31^P NMR of BPMPA.

**Figure S6**. ^1^H NMR of PMB-PMPA.

**Figure S7.** ^13^C NMR of PMB-PMPA.

**Figure S8.** ^13^C-DEPT-135 NMR of PMB-PMPA.

**Figure S9.** ^31^P{^1^H} NMR of PMB-PMPA.

**Figure S10.** ^31^P NMR of PMB-PMPA.

**MDL Method and Calculations for BPMPA and PMB-PMPA**

Prepared stock solutions for BPMPA and PMB-PMPA were prepared, and both benzylated PMPA analytes were combined and added to a concentrated soil extract and diluted this solution with additional soil extract until S/N fell between **5-20**.

- 1. S/N was calculated using extracted ions

                                                               i.      m/z 91 for BPMPA

                                                             ii.      m/z 121 for PMB-PMPA

- 1. S/N was calculated for the **first peak of each doublet**
  2. The spike level for both compounds was 500 ng/mL, but since each compound was split into a doublet, 250 ng/mL as the concentration level to perform the MDL calculations. 7 replicate runs were selected and MDL calculated for both.

In a more detailed manner, here is what we have followed to determine the MDL (LOD) and the LOQ for the method.

We have used the EPA approach for determining the MDL (as this procedure is outlined in the EPA document: *Definition and Procedure for the Determination of the Method Detection Limit, Revision 2*). Specifically, we followed the instructions detailed in the Addendum: Determination of the MDL for a Specific Matrix, and used the specific **OPCW Soil extract** as our “specific matrix.” Reason why we get the ng/mL values.

*EPA Definition and Procedure for the Determination of the Method Detection Limit, Revision 2* – This reference serves as the principle guide for our MDL determination because it directly applies to the analytical approach we used.

*Assigning Values to Non-Detected/Non-Quantified Pesticide Residues in Human Health Food Exposure Assessments (2000)* – This EPA document shows the following calculation: LOQ = 3 x LOD (note: in our paper, we empirically determined a spiking level by preparing a series of dilutions and analyzing the “specific matrix-spiked” samples until the signal-to-noise ratios for the selected quantification ions (m/z 91 for BPMPA and m/z 121 for PMB-PMPA) fell within a range of approximately 10-20). Therefore, we determined the MDL for a specific matrix.

Currie L. A. “Nomenclature in Evaluation of Analytical Methods Including Detection and Quantification Capabilities”. *Pure and Applied Chemistry* **1995,** *67*, 1699-1723. In this document, the author demonstrates and defines the LOQ = 3.04 x DL.
